# Case Report: Metastatic melanoma with initial ctdna decline and radiographic response during self-directed antiparasitic use: treatment effect or spontaneous regression?

**DOI:** 10.3389/fonc.2026.1907192

**Published:** 2026-09-01

**Authors:** Ricky Cheng, Daniel V. Araujo

**Affiliations:** 1 University of Florida College of Medicine, Gainesville, FL, United States; 2 Division of Hematology and Oncology, University of Florida College of Medicine, Gainesville, FL, United States

**Keywords:** case report, circulating tumor DNA, fenbendazole, ivermectin, melanoma

## Abstract

A 74-year-old man with nodular melanoma of the right lateral neck presented with nodal and hepatic metastases at staging. He declined guideline-directed therapy and instead pursued lifestyle modifications alongside self-administered ivermectin and fenbendazole. On serial imaging, his disease initially demonstrated reduced metabolic activity and size without new sites of involvement, paralleled by a decline in tumor-informed ctDNA (Signatera) from 2.04 to 0.18 MTM/mL. Both subsequently worsened, with ctDNA rising to 0.93 MTM/mL and the dominant axillary nodal mass increasing in size and avidity. The tumor carried a mutational burden of at least 50 mutations/Mb, placing it at the extreme upper end of the immunogenic spectrum, and spontaneous immune-mediated regression is at least as plausible an explanation as any effect of the patient’s interventions. The discussion contextualizes the growing popularity of antiparasitic repurposing in oncology, the absence of clinical evidence supporting efficacy, and the need for safety counseling given the reported toxicities of such drugs.

## Introduction

Cutaneous melanoma is an aggressive malignancy with established systemic therapies including immune checkpoint inhibitors (ICIs) and targeted agents, which have improved survival in advanced disease (1). Meanwhile, public interest in repurposed antiparasitics, especially ivermectin and fenbendazole, has increased, driven largely by online narratives and anecdotal reports, despite the absence of clinical evidence in humans. Circulating tumor DNA (ctDNA) is an emerging, minimally invasive biomarker in melanoma; tumor-informed assays complement imaging for tracking disease burden and response (2). To our knowledge, few published reports describe serial PET-CT and ctDNA monitoring in the setting of self-directed antiparasitic use in metastatic melanoma. We report a case of metastatic melanoma with an initial radiographic improvement and ctDNA reduction during self-directed antiparasitic therapy and lifestyle modification.

## Case presentation

A 74-year-old man with hypertension and type 2 diabetes mellitus presented for second-opinion evaluation of advanced melanoma. He was a former smoker (around 40-pack years, quit 5 years prior). He had not received any prior cancer-directed systemic therapy, radiation therapy, or definitive surgical management.

In December 2024, a shave biopsy of a right lateral trapezial neck lesion demonstrated nodular melanoma with Breslow thickness of at least 4 mm, no ulceration identified, and a transected base with involved margins. Subsequent staging PET-CT in January 2025 showed FDG-avid axillary and supraclavicular nodes with a suspicious liver lesion, and an ultrasound-guided biopsy of a large right axillary mass in March 2025 confirmed metastatic melanoma – stage IV [pT4aN2cM1c(0)]. Tumor genomic profiling identified a BRAF V600K mutation, and a high tumor mutation burden (TMB ≥50 mutations/Mb – comprehensive genomic analysis available in the supplement). On initial examination, a palpable supraclavicular node and an approximately 10-cm right axillary nodal mass were noted.

Systemic therapy options were discussed, including immune checkpoint inhibitor regimens, but the patient declined guideline-directed treatments and elected to pursue alternative approaches. Beginning around February 2025, he increased his intake of fruits and vegetables, reduced his intake of processed foods, and aimed to maintain a “neutral” body pH. He also adopted a structured exercise routine consisting of walking 2 miles daily and using a rowing machine. In addition, he self-administered ivermectin (1.87% plunger-tube formulation; approximately “½ inch” of paste or ~ 0.2-0.3 mg/kg per dose) on a 5-days-on/5-days-off schedule and fenbendazole 440 mg once daily on a 10-days-on/7-days-off schedule. He also self-administered a number of other over-the-counter agents including methylene blue, French grape seed extract 150 mg daily, curcumin, berberine, andrographis, milk thistle, a proprietary “liver and gallbladder cleanse,” and vitamin C (5,000 mg daily, later reduced to 3,000 mg). Adherence was not formally assessed but was self-reported. No formal safety monitoring was mandated; however, complete blood counts and a comprehensive metabolic panel were obtained (including hepatic transaminases, alkaline phosphatase, and bilirubin), lactate dehydrogenase, and thyroid function studies at each oncology visit (approximately every 3 months). No clinically significant laboratory abnormalities were identified. He reported no neurologic symptoms, and no focal deficits were noted on examination.

**Figure 1:**
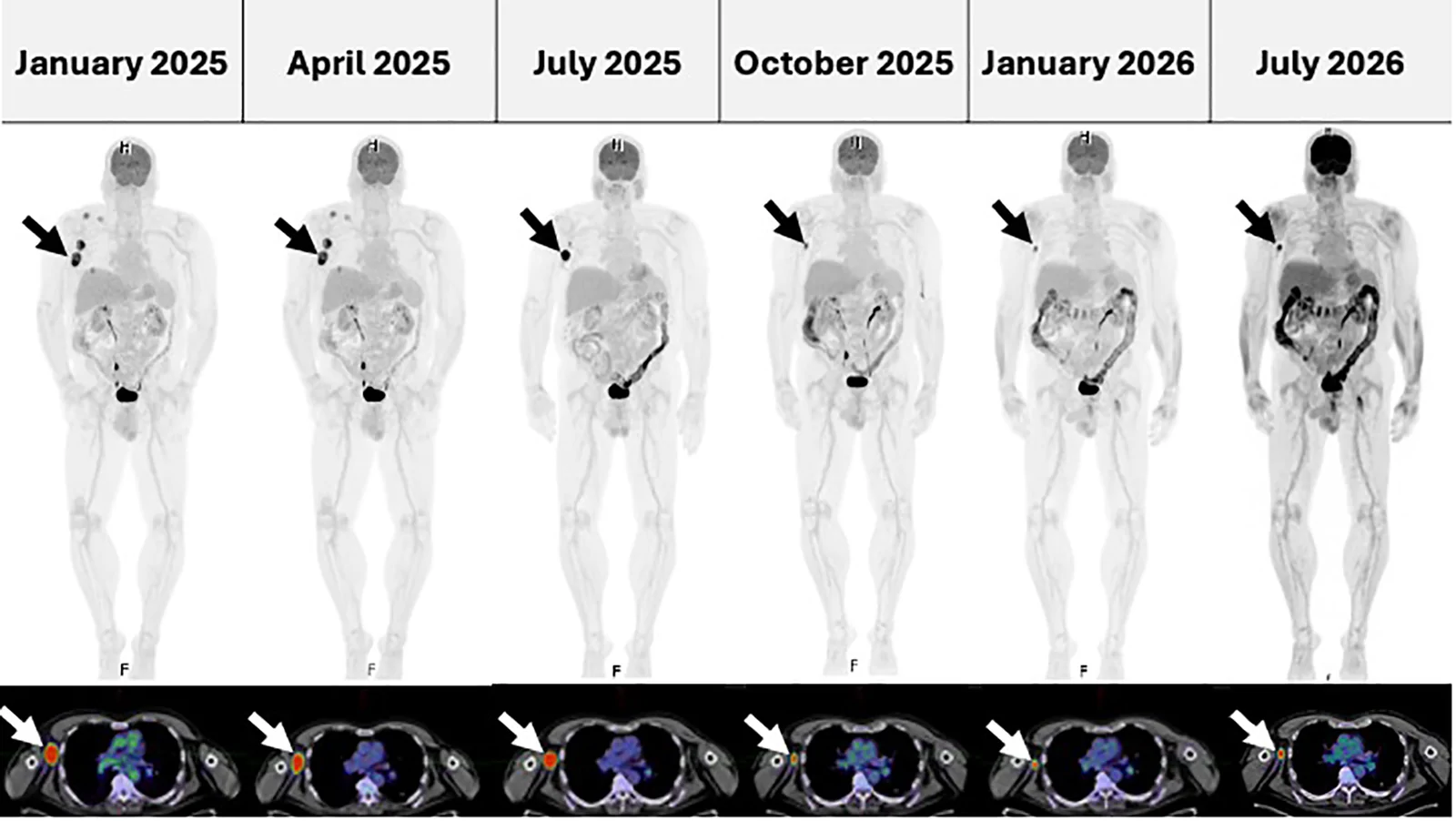
Serial PET-CT scans obtained from January 2025 to July 2026 showing persistent FDG uptake in the right axillary lymph nodes (arrows) on full body projection and corresponding axial images. Baseline SUVmax for the January 2025 study was unavailable. The right axillary lesion SUVmax was 10.8 (April 2025), 13.7 (July 2025), 7.05 (October 2025), and 2.84 (January 2026); the SUVmax for the July 2026 study was unavailable.

**Figure 2:**
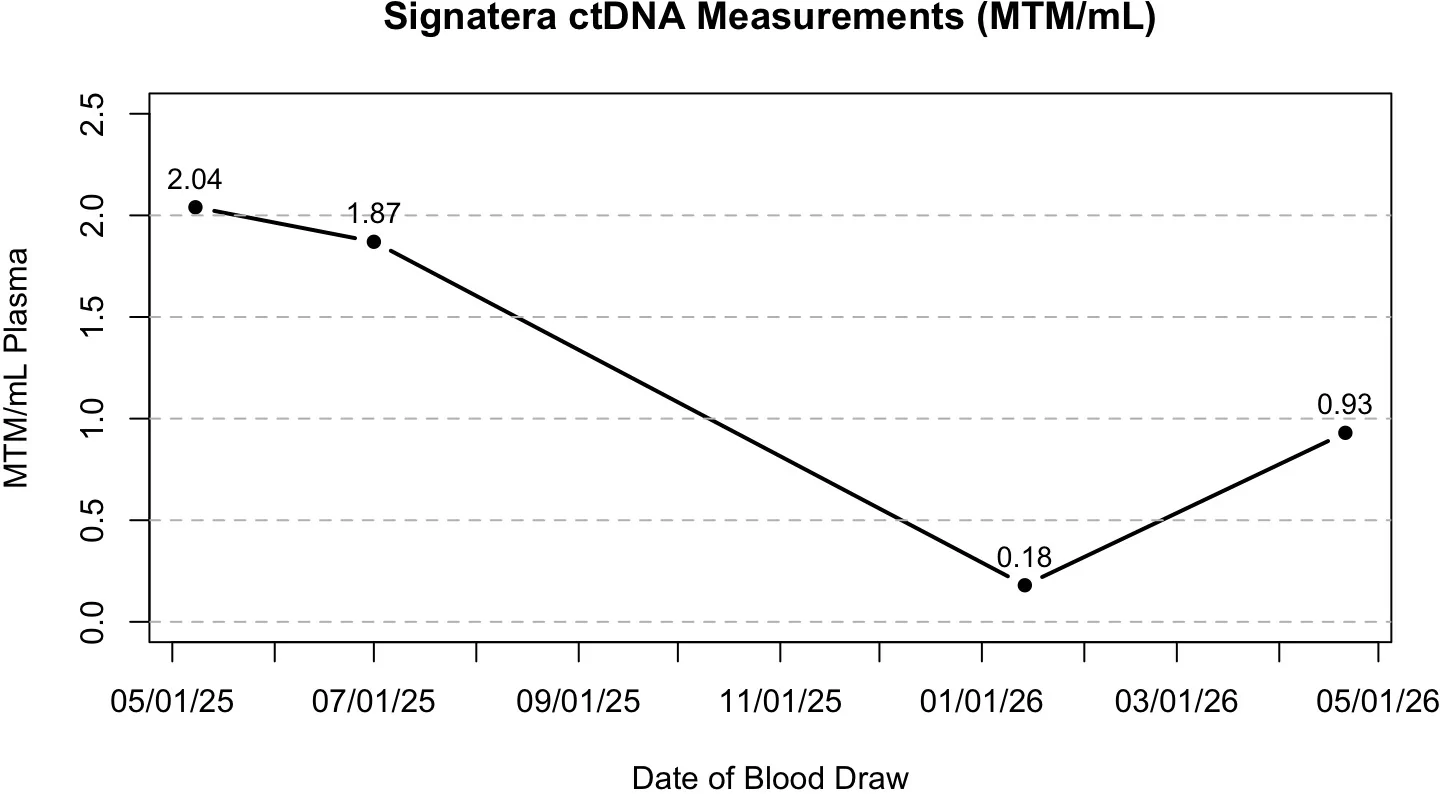
Trend in Signatera ctDNA values over time, showing an initial progressive decrease in plasma ctDNA level from 2.04 MTM/mL to 0.18 MTM/mL between May 2025 and January 2026, followed by a subsequent rise to 0.93 MTM/mL in April 2026. The tumor-informed Signatera assay tracks 16 patient-specific somatic variants selected by whole-exome sequencing of tumor and matched normal tissue, and reports results as mean tumor molecules per mL (MTM/mL). The stated limit of detection is 0.3 MTM/mL, at which analytical sensitivity is 95%.

Disease status was followed with PET-CT (**Figure 1**) and tumor-informed ctDNA (Signatera exome) (**Figure 2**). Signatera ctDNA was 2.04 MTM/mL in May 2025, 1.87 MTM/mL in July 2025, decreased to 0.18 MTM/mL in January 2026, and subsequently increased to 0.93 MTM/mL in April 2026. Six PET-CT studies were obtained between January 2025 and July 2026 (**Figure 1**). PET-CT in April 2025 showed persistent FDG-avid right axillary adenopathy. PET-CT in July 2025 showed a mixed response (one right axillary node increased in size and avidity, while adjacent axillary and supraclavicular nodes decreased). PET-CT in October 2025 demonstrated interval decrease in FDG avidity and size of the right axillary nodes. PET-CT in January 2026 showed further reduction in avidity, grossly stable in size, without definitive progression or new disease. The most recent PET-CT, in July 2026, showed both an increase in size and FDG uptake in his axillary node. Overall, imaging and ctDNA initially improved, but subsequently worsened. To date, the patient remains clinically well and continues to decline standard systemic therapies. He is open to axillary dissection, which is being discussed. He will continue to undergo restaging imaging and ctDNA monitoring.

## Discussion

This case describes an initial improvement in metabolic imaging and ctDNA in a patient with metastatic melanoma who declined guideline-directed systemic therapy and instead pursued lifestyle modifications with self-administered ivermectin and fenbendazole. Although a temporal association between these interventions and disease regression is observed, the findings arise from an uncontrolled setting and should be interpreted as descriptive rather than indicative of causation. Concurrent lifestyle changes and the use of multiple supplements represent potential confounders and preclude attribution of improvement to any single intervention.

Furthermore, melanoma has the highest rate of spontaneous regression among solid tumors, approximately six times that of other malignancies, although spontaneous regression in metastatic disease remains exceedingly rare (0.2%-0.3%) (3–6). Notably, this patient’s tumor mutational burden (TMB) exceeded 50 mutations/Mb, far surpassing the median reported for melanoma (13-14 mutations/Mb) and placing the tumor at the extreme upper end of the immunogenic spectrum (7, 8). Higher TMB is hypothesized to increase neoantigen burden and consequently enhance anti-tumor immunogenicity. Within a given tumor histology, higher TMB is associated with improved outcomes following ICI treatment (7, 9–12). Taken together, these findings support spontaneous immune-mediated tumor regression as a plausible explanation independent of any intervention.

Off-label antiparasitic regimens have been promoted in public forums as potential anticancer strategies, often supported by anecdotal reports rather than clinical evidence. Concurrently, the treatment landscape for advanced melanoma has been transformed by evidence-based systemic therapies, including immune checkpoint inhibitors and targeted agents, which have improved response rates and survival for many patients (1). For BRAF V600-mutant stage IV disease such as this patient’s, first-line nivolumab plus ipilimumab achieves 10-year overall survival of approximately 43% (13) —in other words, ICIs cure more than 40% of patients with metastatic melanoma, a figure that would have been unthinkable in the chemotherapy era, when fewer than 10% of patients achieved 5-year overall survival. For patients with contraindications to ICIs, or who prefer not to receive them, BRAF/MEK inhibitor combinations produce response rates exceeding 60% with rapid disease control in tumors harboring BRAF V600E/K mutations, albeit with inferior long-term survival compared with upfront ICI (14). As these parallel alternative treatment narratives increasingly intersect in clinical practice, clinicians will increasingly need to counsel patients who defer standard therapy about the survival benefit they are forgoing.

Both ivermectin and fenbendazole have plausible mechanistic hypotheses and preclinical signals (15–17). Ivermectin has demonstrated anticancer effects across a range of *in vitro* models and, in selected studies, suppressed tumor growth in xenograft models (16–18). Fenbendazole has been reported to exert anticancer activity in experimental systems, including effects consistent with microtubule disruption in mammalian cells (15). However, the existence of preclinical activity does not establish clinical benefit, particularly when human dosing, pharmacokinetics, tumor microenvironment, immune interactions, and endpoints differ substantially from bench conditions. Notably, most preclinical studies demonstrating anticancer activity have required micromolar concentrations that substantially exceed the plasma levels achievable with approved human dosing, further limiting direct clinical translation (19). Consistent with this translational gap, contemporary reviews emphasize that there is currently no clinical evidence demonstrating anticancer efficacy of ivermectin or fenbendazole in humans, and no randomized trials have shown therapeutic benefit (18, 20). Accordingly, this case should be interpreted at best as hypothesis-generating rather than providing evidence of therapeutic effect.

More recently, ctDNA levels have risen, and PET-CT demonstrated both an increase in size and FDG uptake of the axillary mass. Together, these findings are consistent with disease progression. The interval of improvement with this alternative approach was therefore limited (approximately 11 months), standing in contrast to the more durable disease control typically observed with ICIs (13). This further underscores the urgent need to rigorously investigate these compounds and define what they can and cannot do, so that patients can be counseled accurately.

Even when patients decline guideline-directed therapy, clinicians can continue to provide meaningful care through harm-reduction counseling, shared decision-making, and appropriate longitudinal monitoring. Maintaining a therapeutic alliance is essential to ensure patient safety and to mitigate preventable adverse effects. Safety concerns related to these agents are not merely theoretical. Ivermectin toxicity has been described, particularly in the setting of supratherapeutic dosing and nonstandard formulations or routes of administration, and may include significant neurotoxicity (21, 22). Published case reports have also described fenbendazole-associated drug-induced liver injury following self-administration (23, 24). Fenbendazole is not approved for human use, and human pharmacokinetic data are correspondingly sparse, so neither an effective nor a safe human dose can be defined. Additionally, both ivermectin and fenbendazole possess antimicrobial properties and may theoretically alter gut microbiome composition. Disruption of the microbiome has been associated with impaired outcomes in patients receiving immune checkpoint inhibitors, particularly following antibiotic exposure (25). This evidence derives from patients receiving ICIs and does not translate directly to a patient on no systemic therapy; nevertheless, given that melanoma is a highly immunogenic tumor in which endogenous immune control is the most plausible driver of the observed regression, the potential for unintended immunologic consequences warrants caution.

Given the absence of proven clinical benefit and the possibility of harm, we strongly discourage the use of unproven off-label medications outside of a clinical trial. At the same time, we recognize that patient interest in these agents reflects a broader need for rigorous scientific evaluation. Generating high-quality evidence is preferable to allowing therapeutic decisions to be driven by anecdote. One example of such efforts is the phase I/II study by Yuan et al., evaluating ivermectin in combination with balstilimab (anti–PD-1) in patients with triple-negative breast cancer (20). As a scientific and clinical community, we have a responsibility to systematically investigate questions that are meaningful to our patients, while upholding standards of safety and evidence-based practice.

## Limitations

This report is limited by its single-patient design, lack of a comparator, uncertain adherence and exact dosing, and the possibility of unmeasured confounders. Because adherence was self-reported, the temporal association between the reported regimen and the observed clinical course remains uncertain. The observation should therefore be viewed as hypothesis-generating at best, underscoring the need for rigorous clinical research.

## Conclusion

In this case of metastatic melanoma in which standard systemic therapy was deferred, serial PET-CT imaging and tumor-informed ctDNA exhibited an initial decrease during self-directed antiparasitic use and lifestyle modification, though ctDNA subsequently rose and imaging progressed, indicating that any period of improvement was limited and transient. While the longitudinal trend is notable, the observation remains non-controlled and does not support causal attribution or clinical efficacy. Given the absence of human evidence for anticancer benefit and the reported adverse events, clinicians should counsel patients on uncertainty, document nonstandard exposures, and consider safety monitoring (including liver function testing when fenbendazole is used). Further research is warranted to characterize outcomes and toxicities and to determine whether any reproducible association exists in controlled clinical settings.

## Data Availability

The original contributions presented in the study are included in the article/**Supplementary Material**. Further inquiries can be directed to the corresponding author.

## Supplementary material

Supplementary Figure 1 Tumor genomic profiling from Natera demonstrating genomic alterations, tumor mutation burden, microsatellite status, PD-L1 expression, and additional significant alterations.
