# Supplementary material for: Case Report: Metastatic melanoma with initial ctdna decline and radiographic response during self-directed antiparasitic use: treatment effect or spontaneous regression?
Source: Front Oncol. 2026 Sep 1;16:1907192. doi: 10.3389/fonc.2026.1907192 (PMC13574632; doi:10.3389/fonc.2026.1907192)
Supplement: Supplementary file 1 [file DataSheet1.pdf]

CARE case report guidelines

CARE Checklist of information to include when writing a case report

(cc) BY-NC-ND

Topic	Item	Checklist item description	Reported on Line
Title	1	The diagnosis or intervention of primary focus followed by the words "case report"	1-2
Key Words	2	2 to 5 key words that identify diagnoses or interventions in this case report, including "case report"	32
Abstract (no references)	3a	Introduction: What is unique about this case and what does it add to the scientific literature?	26-30
	3b	Main symptoms and/or important clinical findings	19-22
	3c	The main diagnoses, therapeutic interventions, and outcomes	19-25
	3d	Conclusion—What is the main "take-away" lesson(s) from this case?	26-30
Introduction	4	One or two paragraphs summarizing why this case is unique (**may include references**)	35-48
Patient Information	5a	De-identified patient specific information.	51-57
	5b	Primary concerns and symptoms of the patient.	51-52;59-68
	5c	Medical, family, and psycho-social history including relevant genetic information	52-57;64-66
	5d	Relevant past interventions with outcomes	56-57
Clinical Findings	6	Describe significant physical examination (PE) and important clinical findings.	66-68
Timeline	7	Historical and current information from this episode of care organized as a timeline	59-91
Diagnostic Assessment	8a	Diagnostic testing (such as PE, laboratory testing, imaging, surveys).	59-66;83-89
	8b	Diagnostic challenges (such as access to testing, financial, or cultural)	66
	8c	Diagnosis (including other diagnoses considered)	64-66
	8d	Prognosis (such as staging in oncology) where applicable	64-66
Therapeutic Intervention	9a	Types of therapeutic intervention (such as pharmacologic, surgical, preventive, self-care)	70-79
	9b	Administration of therapeutic intervention (such as dosage, strength, duration)	72-79
	9c	Changes in therapeutic intervention (with rationale)	N/A
Follow-up and Outcomes	10a	Clinician and patient-assessed outcomes (if available)	83-91
	10b	Important follow-up diagnostic and other test results	83-91
	10c	Intervention adherence and tolerability (How was this assessed?)	79-81
	10d	Adverse and unanticipated events	80-81
Discussion	11a	A scientific discussion of the strengths AND limitations associated with this case report	94-99; 155-159
	11b	Discussion of the relevant medical literature **with references**.	35-48; 103-153
	11c	The scientific rationale for any conclusions (including assessment of possible causes)	94-153
	11d	The primary "take-away" lessons of this case report (without references) in a one paragraph conclusion	162-170
Patient Perspective	12	The patient should share their perspective in one to two paragraphs on the treatment(s) they received	N/A
Informed Consent	13	Did the patient give informed consent? Please provide if requested	Yes [X] No [ ]
